# Neurocysticercosis Diagnosis in a Non-Endemic Country: France

**DOI:** 10.3390/pathogens12101205

**Published:** 2023-09-29

**Authors:** Ines Zemmour, Marie-Fleur Durieux, Etienne Herault, Célia Rouges, Barbara Šoba, Aurélien Mercier, Frédéric Ariey, Pierre-Marie Preux, Hélène Yera, on behalf of Collaborators Group

**Affiliations:** 1 Parasitology-Mycology Laboratory, Dupuytren Universitary Hospital Center, 87000 Limoges, France; ines.zemmour@chu-limoges.fr (I.Z.); marie-fleur.durieux@chu-limoges.fr (M.-F.D.); etienne.herault@unilim.fr (E.H.); 2 Inserm U1094, IRD UMR270, University of Limoges, CHU Limoges, EpiMaCT—Epidemiology of Chronic Diseases in the Tropical Zone, Institute of Epidemiology and Tropical Neurology, OmegaHealth, 87000 Limoges, France; aurelien.mercier@unilim.fr (A.M.); pierre-marie.preux@unilim.fr (P.-M.P.); 3 Parasitology-Mycology, Hôpital Cochin, APHP, Université de Paris, INSERM 1016, Institut Cochin, 75014 Paris, France; celia.rouges@aphp.fr (C.R.); frederic.ariey@aphp.fr (F.A.); 4 Laboratory of Parasitology, Institute of Microbiology and Immunology, Faculty of Medicine, University of Ljubljana, 1000 Ljubljana, Slovenia; barbara.soba@mf.uni-lj.si

**Keywords:** neurocysticercosis, *Taenia solium*, diagnosis, PCR, serology, non-endemic countries, Europe, epilepsy

## Abstract

Diagnosing neurocysticercosis (NCC) is difficult due to its variable clinical presentations and the different imaging techniques used to detect brain damage. This study aimed to evaluate the use of cerebrospinal fluid serology and PCR for diagnosing biological neurocysticercosis in a non-endemic country. We tested samples from patients living in France with suspected NCC and confirmed that 45 of the patients presented with the disease. A total of 89% of patients had previously traveled to countries where the disease was endemic. The sensitivity of Western blots compared to ELISA was not significantly different (80% vs. 60%) (*p* > 0.05), and neither was the sensitivity of Western blots vs. PCR (78% vs. 56%) (*p* > 0.05). The PCR sensitivity was 78% and 47% in definitive NCC and in probable NCC. PCR tests using cerebrospinal fluid should be considered as a diagnostic criterion for identifying NCC.

## 1. Introduction

Neurocysticercosis (NCC) is a parasitic disease of the central nervous system caused by *Taenia solium* larvae in the brain [1]. Cysticercosis is considered the most frequent parasitic disease of the central nervous system in the world, with significant levels of prevalence in Central and South America, India, East Asia, Eastern Europe, and Africa, though with the exception of Muslim countries [2]. Local populations use farm pigs as sources of cheap and readily available meat, but despite prevention and control efforts, the disease continues to be a major health problem in these regions [3,4]. Moreover, the prevalence of this disease is higher in developing regions, where sanitation and wastewater treatment practices are often inadequate [3].

NCC is a major cause of parasitic epilepsy worldwide [5], causing 30–50% of epileptic seizures in endemic countries [5,6]. In NCC, seizures represent the main clinical manifestations and are described in 79% of cases of patients with symptomatic disease [7]. NCC can also lead to other neurological disorders, such as intracranial hypertension, neurological deficits, and mental disorders [8]. Symptoms may be mild or severe, depending on the location, stage, and number of larvae or cysts in the brain.

Due to its variable clinical presentations and the multitude of diagnostic tests available, NCC can be difficult to diagnose [1]. Diagnosis is mainly based on the use of imaging techniques, such as computed tomography (CT) and magnetic resonance imaging (MRI), which are expensive and rarely accessible in the most-affected areas [8].

In addition to imaging techniques, serological tests using cerebrospinal fluid (CSF), ELISA, and Western blots (WBs) can detect *T. solium* antigens or antibodies to the parasite [9]. They are generally used to confirm the diagnosis of NCC in patients with suspicious brain lesions, and they are accessible and inexpensive diagnostic tools in endemic areas [8]. However, serology may lack specificity [4,8,9]. When using crude antigens, cross-reactions are possible with other helminthiases (such as cystic echinococcosis and hymenolepiasis). ELISA has been shown to have lower specificity than WBs when using CSF (90% vs. 100%) [9]. Moreover, 10–20% of individuals in endemic populations may present specific antibody reactions due to viable NCC infections, but this may also be due to infections outside the central nervous system, passive transfer from their mothers, exposure without infection, or infections that had resolved spontaneously [4].

Confirmation of the diagnosis may require a brain biopsy with a histological study. This invasive procedure should be used with caution.

With regard to molecular techniques, a polymerase chain reaction (PCR) test using CSF is a sensitive and specific technique for diagnosing NCC [10]. It also has the advantage of being less invasive than a brain biopsy and less expensive than imaging.

This paper presents a retrospective study of NCC diagnosis in a non-endemic area and evaluates the available diagnostic techniques.

## 2. Materials and Methods

### 2.1. Samples

In France, the biological diagnosis of cysticercosis using blood, CSF, and tissue is only performed in some centers [11]. The parasitology-mycology laboratories of the university hospitals of Cochin and Limoges carry out the detection of parasite DNA using molecular PCR techniques and the detection of antibodies using ELISA or WBs.

The results of samples received in these two hospitals between 2004 and 2022 were collected. Serology or PCR-positive samples for cysticercosis were selected. Then, corresponding clinical, epidemiological, and radiological information was retrieved.

The clinical data included medical histories, neurological symptoms, and general clinical signs. The epidemiological data included information on travel, contact with infected persons, and exposure to potential sources of contamination. Radiological data were obtained using imaging techniques, such as CTs and MRIs, to identify the morphological features, shapes, and locations of brain lesions.

### 2.2. T. solium PCR

The *T. solium* PCR technique was performed as described by Yera et al. [10], except the probe was modified in one center as 5′-6-carboxyfluorescein-GCAGTCCACACGGCAAAGGACA-black hold quencher-3′. Briefly, DNA was extracted from 1 mL of serum or 200–500 µL of CSF using the biological fluid protocol of the QIAmp DNA mini kit (Qiagen) or from 25 mg of cerebral biopsy using the tissue protocol of the same kit. DNA samples were eluted in 100 µL of sterile distilled water. The DNA amplification targeted the pTsol9 repetitive element of the parasite’s nuclear genome using TaqMan probe detection. PCR inhibition was checked in each DNA extract by amplifying a non-competitive internal control (IC) in an additional reaction for a real-time PCR assay. The absence of amplification of the IC was considered a significant inhibition.

### 2.3. Cysticercosis Serology

#### 2.3.1. ELISA

Determination using IgG in serum and CSF samples was performed by ELISA using a *Taenia solium* IgG^®^ kit (NovaLisa NovaTec, Launch Diagnostics, 75008 Paris, France) according to the manufacturer’s recommendations in only one center. Purified antigens were prepared from *T. solium* lysates. According to the French nomenclature of medical biology acts, positive or equivocal results from ELISA were confirmed with WBs.

#### 2.3.2. Western Blot

The Western blot detection of IgG in serum and CSF was performed using a Cysticercosis Western Blot IgG^®^ kit (LDBIO Diagnostics, 69009 Lyon, France) according to the manufacturer’s recommendations in both centers. It consisted of ready-to-use nitrocellulose strips onto which *T. solium* antigens (i.e., porcine cysticerci extracts) were transferred after electrophoretic migration on a polyacrylamide gel. A WB was considered positive if at least two bands were present in P6-8, P12, P23-26, P39, and P50-55 [12].

### 2.4. Criteria for Defining NCC

We used the criteria set out by Del Brutto to define and classify NCC cases [8]. These criteria considered several clinical, radiological, histological, immunological, and epidemiological elements of the patients. They included four diagnostic categories: absolute, major, minor, and epidemiological. Interpretation of these criteria allowed for two degrees of diagnostic certainty, definitive and probable, depending on the likelihood that NCC was present in a given patient.

### 2.5. Statistical Analysis

The sensitivities of the biological methods were compared using Fisher’s exact test. A probability of 0.05 or less was considered to be significant.

## 3. Results

A total of 1145 samples were received for cysticercosis diagnosis: 616 and 529 in the laboratories of Cochin and Limoges university hospitals, respectively. One hundred and six samples (9%) were positive for cysticercosis using serology or PCR, with two biological arguments in favor of cysticercosis. These samples were from 45 patients for whom clinical, radiological, and epidemiological information and serological and molecular results were collected (Appendix A).

### 3.1. Clinical, Radiological, and Epidemiological Data

Most patients (82%; 37 of 45) had symptoms and lesions suggestive of NCC.

The various symptoms were headaches, seizures, psychiatric and visual disturbances, focal neurological deficits, meningitis, and meningoencephalitis. In the study population, the prominent neurological manifestations observed were epilepsy (53% of cases; 19 of 36) and headaches (39% of patients; 14 of 36).

The brain imaging results indicated the possibility of NCC as follows: 28% (11 of 39) of cases exhibited cysts, 23% (9 of 39) had calcification, and 21% (8 of 39) showed signs of hydrocephalus. In addition, 8% of cases (3 of 39) presented with ventricular dilatation. These results emphasized the diverse range of radiological symptoms detected in the patient group.

Most patients (89%; 33 of 37) had traveled to countries where the disease was endemic. The remaining four cases (C9, C14, C15, and C36) were contaminated in Europe (Portugal, Bosnia and Herzegovina, Serbia, and France, respectively). The French case, C36, had no history of foreign travel or contact with infected persons, but he had consumed raw pork sausages. In France, pork farming is controlled, which protects against cysticercosis and trichinellosis. However, the origin of the meat pork was not available, and we could not exclude that it was imported or came from French traditional breeding.

### 3.2. Serology and PCR

The ELISA results were positive in 7 of 11 CSF (64%) and 10 of 17 sera samples (59%) for the 45 suspected cases of NCC (Table 1).

The WB tests were positive in 28 of 36 CSF (78%) and 27 of 30 sera (90%) samples. The PCR tests were positive in 34 of 64 samples (53%) and precisely positive in 0 of 12 sera samples (0%), 30 of 48 CSF samples (63%), and all biopsies (100%).

We compared the performances of ELISA and WB tests in detecting specific antibodies in the same CSF or sera samples. In 10 CSF samples, 6 were positive and 4 were negative when ELISA tests were used whereas 8 were positive and only 2 were negative when WBs were used. In 15 sera samples, 9 were positive and 6 were negative when ELISA tests were used whereas 13 were positive and only 2 were negative when WBs were used. The ELISA and WB sensitivities were not significantly different for the CSF (60% vs. 80%) or sera (60% vs. 87%; *p* > 0.05) samples.

We also compared the sensitivities of WB and PCR tests in detecting NCC in the same CSF samples. In 32 CSF samples, 25 were positive, 4 were negative, and 3 were borderline when WBs were used whereas 18 were positive and 14 were negative when PCR tests were used. The PCR and WB sensitivities were not significantly different (56% vs. 78%; *p* > 0.05).

### 3.3. Classification of Cases according to Del Brutto’s Criteria

According to the criteria established by Del Brutto [8] 21 patients had definitive NCC diagnoses while 15 had probable diagnoses and 9 patients were unclassifiable.

**Figure 1:**
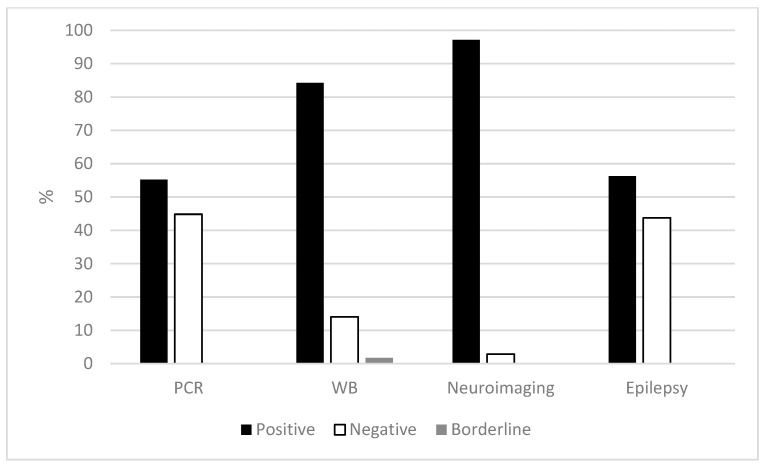
Principal diagnostic elements of cases with definitive or probable NCC diagnoses.

For the diagnostic elements in definitive or probable NCC, the positivity rates were higher in neuroimaging (97%), followed by WB (84%) then by epilepsy (56%) and by PCR (55%) (Figure 1).

For CSF samples, the WB and PCR tests had detection rates of 79% (23 of 29) and 67% (28 of 42), respectively (Table 2).

The ELISA results are not presented because the number of samples tested was low.

When comparing the PCR and WB performances on the same samples, 16 of 26 CSF samples were positive and 10 were negative when PCR tests were used whereas 21 were positive, 4 were negative, and 1 was borderline when WBs were used. For the CSF samples, the sensitivities of the PCR and WB tests were not significantly different (62% vs. 81%; *p* > 0.05). When combining the WB and PCR results for the CSF samples, the sensitivity of NCC diagnosis increased to 85% (22 of 26).

The PCR results for the CSF samples showed 78% and 47% sensitivities for definitive and probable NCC, thus confirming the NCC diagnoses.

All biopsies were positive when PCR tests were used.

## 4. Discussion

In the diagnosis of NCC, the patient clinical presentations and the performances of available diagnostic tests are variable. A combination of imaging techniques, serological testing, and histology can be used to reliably diagnose NCC in patients with suspicious brain lesions. Imaging techniques, such as CTs and MRIs, are commonly used to detect lesions in the brain, but these methods may not be specific to NCC. In addition, CTs may provide false-negative results for calcified cysts and MRIs may lack sensitivity for detecting small cysts. Serology can be used to detect antibodies to *T. solium* antigens, but it can also produce false-positive or negative results, especially in patients with extraneural forms of the disease [8,9]. Histology, which provides a reliable diagnosis, is invasive. These limitations can render the diagnosis of NCC difficult [13].

In addition, the lack of financial means allows only a limited part of the world population to access complementary examinations, such as imaging [1]. Therefore, clinical judgments and basic laboratory tests are generally required to investigate NCC in developing countries.

In this context, PCR using CSF samples has emerged as a sensitive and specific molecular diagnostic technique for diagnosing NCC. PCR tests can detect *T. solium* DNA in the CSF samples of NCC patients with high sensitivity and specificity. We confirmed that PCR using CSF samples and brain biopsies has shown good sensitivity (79% and 56% in definitive and probable NCC, respectively). However, PCR may lack sensitivity for parenchymal versus extra-parenchymal lesions [14] or in cases if single and calcified lesions [10]. Thus, in our study, the PCR results were negative for the CSF samples of 10 patients with confirmed NCC, and among them, 80% (8 of 10) had single intraparenchymal lesions, of which 25% (2 of 8) were calcified. Diagnoses were confirmed by serology for CSF (4 of 7) or sera (5 of 5) samples or by PCR tests using brain biopsies (3 of 3). When examining the origins of contamination (one from Bolivia, one from Cameroon, one from Cape Verde, one from DRC, three from India, one from Madagascar, and one from Portugal), the CSF samples with negative PCR results were not related to a particular region.

PCR testing is not widely available in areas where NCC is prevalent, and it is often considered an expensive and complex diagnostic method. Therefore, additional efforts are needed to improve access to PCR testing and evaluate its application in different healthcare settings.

In France, routine ELISA screening and confirmation by WB could miss positive results due to false-negative ELISA results. In this study, WBs exhibited higher levels of sensitivity for CSF samples than ELISA tests, but the difference was not significant (probably due to a low sampling). Nevertheless, we recommend performing Western blot and PCR tests on CSF samples in cases where NCC is strongly suspected.

In Europe, NCC is generally considered an imported disease as it occurs in individuals who have traveled to or lived in endemic areas, including Central and South America, India, and East Asia. Travelers or migrants from these regions may carry *T. solium* larvae in their central nervous systems [15]. A review of the literature revealed numerous cases of NCC diagnoses in various European countries, and particular epidemic situations were highlighted in Spain and Portugal [16]. Although isolated cases of NCC have been reported in Slovenia, the prevalence of this disease is considered low and the cases are due to immigration from countries of the former Yugoslavia [17]. In addition to *T. solium*, other types of tapeworms can also cause NCC. A case of cysticercosis-like cerebral infection caused by the tapeworm *Taenia martis* was documented in a French patient who had not traveled outside Europe [18]. This species is usually found in adult stages in the small intestines of stone martens.

It is essential to raise awareness about NCC among healthcare professionals, even in Europe, and to improve knowledge of the disease to ensure early diagnosis and appropriate treatment. In addition, preventive measures, such as informing travelers and monitoring potentially contaminated food imports, can help reduce the incidence of NCC in Europe.

The criteria established by Del Brutto are used to define and classify NCC cases [8]. However, they do not include PCR tests using CSF samples or brain biopsies, although these could be considered as absolute diagnostic criteria. Future studies should evaluate the diagnostic value of parasitic DNA detection in CSF samples or brain biopsies using latent class analysis (LCA) to study the performance of different available methods in the absence of an absolute gold standard or to reduce the bias of an imperfect gold standard [19,20].

## Figures and Tables

**Table 1:** Serology and PCR results of the cysticercosis cases.

	Result	Serum	CSF (^a^)
ELISA	positive	10	7
	negative	7	4
WB	positive	27	28
	negative	3	5
	borderline	0	3
PCR	positive	0	30 (4)
	negative	12	18

^a^ Brain biopsy.

**Table 2:** Serology and PCR results of definitive and probable cysticercosis cases.

	Result	Definitive NCC Serum/CSF (^a^)	Probable NCC Serum/CSF (^a^)
WB	positive	17/12	6/11
	negative	1/2	2/3
	borderline	0/1	0/0
PCR	positive	0/21 (1)	0/7 (3)
	negative	12/6	0/8

^a^ Brain biopsy.

## Data Availability

Data availability is limited due to ethical restrictions.

## Appendix A. Clinical, Radiological, Epidemiological, Serological, and Real-Time PCR Results of Cases

**Case**	**Clinical Signs**	**Cerebral Radiology Result**	**Epidemiology**	**No. of Samples**	**Serological Result** **ELISA** **Western-Blot**		**Real-Time PCR Result**	**Del Brutto [8] Classification**
C1	No information	No information	Togo	1 serum	Serum negative/CSF Nd	Serum positive/CSF Nd	Nd	Unclassified
C2	No information	No information	Togo	1 serum, 1 CSF	Serum positive/CSF positive	Serum positive/CSF positive	Nd	Probable NCC
C3	Epilepsy	1 cystic lesion in the frontal lobe	Haiti	1 serum, 1 CSF	Serum negative/CSF negative	Serum positive/CSF negative	Nd	Definitive NCC
C4	No information	No information	No information	1 serum, 1 CSF	Serum positive/CSF negative	Serum positive/CSF positive	Nd	Unclassified
C5	No information	No information	No information	1 serum	Serum negative/CSF Nd	Serum positive/CSF Nd	Nd	Unclassified
C6	Headaches, visual disorders	5 cysts with scolex and perilesional edema	Madagascar	1 serum	Serum negative/CSF Nd	Serum positive/CSF Nd	Serum negative/CSF Nd	Definitive NCC
C7	Epilepsy	4 bilateral temporal lesions	Travel Ivory Coast, Chad	1 serum	Serum negative/CSF Nd	Serum positive/CSF Nd	Nd	Definitive NCC
C8	No information	No information	No information	1 serum, 1 CSF	Serum positive/CSF negative	Serum positive/CSF positive	Serum Nd/CSF positive	Unclassified
C9	Epilepsy, disorders of consciousness, neurological signs	1 calcification	Portugal	1 serum, 2 CSF	Nd	Serum positive/CSF positive	Serum Nd/CSF negative X2	Probable NCC
C10	Visual blur, headaches, epilepsy	Cerebral lesions	No information	1 serum, 1 CSF, 1 brain biopsy	Serum positive/CSF Nd	Serum positive /CSF Nd	Serum Nd/CSF negative/ brain biopsy positive	Probable NCC
C11	Chronic meningoencephalitis	No anomaly found	Madagascar	1 serum, 1 CSF	Serum positive/CSF positive	Serum positive/CSF positive	Serum Nd/CSF positive	Probable NCC
C12	Chronic meningitis, intracranial hypertension	2 microcalcifications (occipital region)	Guatemala, NCC in 2007	1 serum, 1 CSF	Serum positive/CSF positive	Serum Nd/CSF positive	Nd	Probable NCC
C13 *	No information	1 abscess	India	1 serum, 1 CSF, 1 brain biopsy	Nd	Nd	Serum negative/CSF negative/brain biopsy positive	Definitive NCC
C14	Headaches, mental changes, basal meningitis	1 cyst above planum sphenoidal, small frontotemporal cysts, hydrocephalus, arachnoiditis	Bosnia and Herzegovina	1 serum, 1 CSF	Serum positive/CSF positive	Serum positive/CSF positive	Serum Nd/CSF positive	Definitive NCC
C15	Mental changes	Hydrocephalus, 1 intraventricular cyst	Serbia	1 serum, 1 CSF	Serum positive/CSF positive	Serum positive/CSF positive	Serum Nd/CSF positive	Definitive NCC
C16	Epilepsy	1 calcified lesion, multiple intracerebral lesions	Congo	1 serum, 2 CSF	Nd	Serum negative/CSF negative X2	Serum Nd/CSF negative then positive	Probable NCC
C17	Meningitis, neurological signs	1 active arachnoidal ventricular lesion	Guyane, Haiti	1 CSF	Nd	Serum Nd/CSF positive	Serum Nd/CSF positive	Probable NCC
C18	Behavioral disorder, mutism	Tetraventricular dilatation, hydrocephalus	Madagascar	1 serum, 1 CSF	Nd	Serum positive/CSF positive	Serum Nd/CSF positive	Probable NCC
C19	Chronic meningitis, right hemiparesis	Normal scanner	Cape Verde	1 CSF	Nd	Serum Nd/CSF positive	Serum Nd/CSF positive	Unclassified
C20	Headaches, chronic meningitis	Cysts	Haiti	1 serum, 2 CSF	Nd	Serum positive/CSF positive X2	Serum Nd/CSF positive X2	Definitive NCC
C21	Eosinophilic meningitis, headache	1 brain lesion, 1 calcification	Mexico	1 CSF	Nd	Serum Nd/CSF positive	Serum Nd/CSF positive	Probable NCC
C22 *	No information	Compression and bypass of the 4th ventricle	No information	1 CSF	Nd	Nd	Serum Nd/CSF positive	Definitive NCC
C23	No information	1 intracranial lesion, 1 cyst, hydrocephalus	Cape Verde	1 CSF	Nd	Serum Nd/CSF positive	Serum Nd/CSF positive	Probable NCC
C24	Epilepsy	Multiple cystic lesions (12)	Congo	1 serum, 1 CSF	Nd	Serum positive/CSF Nd	Serum Nd/CSF positive	Definitive NCC
C25	Chronic meningitis	Lepto-meningitis	No information	1 CSF	Nd	Serum Nd/CSF limit	Serum Nd/CSF negative	Unclassified
C26	Hemiplegia, epilepsy	1 cerebral lesion with per-lesion edema	DRC	2 CSF	Serum Nd/CSF positive	Serum Nd/CSF positive X2	Serum Nd/CSF negative X2	Probable NCC
C27	Epilepsy	Multifocal cysts	No information	1 CSF	Nd	Serum Nd/CSF positive	Serum Nd/CSF negative	Unclassified
C28	No information	No information	Cape Verde	1 CSF	Nd	Serum Nd/CSF positive	Serum Nd/CSF negative	Unclassified
C29 *	Temporal headaches	1 cyst	Bolivia	1 serum, 1 CSF	Nd	Serum Nd/CSF limit	Serum negative/CSF negative	Definitive NCC
C30	Headaches	1 frontal cystic lesion, perilesional edema	No information	1 CSF	Nd	Serum Nd/CSF limit	Serum Nd/CSF negative	Unclassified
C31	Epilepsy	1 active and inflammatory lesion	Madagascar	1 CSF	Nd	Serum Nd/CSF positive	Serum Nd/CSF negative	Probable NCC
C32	Epilepsy, intracranial hypertension, motor deficit	1 calcified frontal cortico-subcortical nodular lesion	India	1 serum, 1 CSF	Nd	Serum positive/CSF Nd	Serum Nd/CSF negative	Definitive NCC
C33	Epilepsy	1 left temporal cerebral lesion	Cameroon	1 serum, 1 CSF, 1 brain biopsy	Serum negative/CSF negative	Serum Nd/CSF negative	Serum Nd/CSF negative/brain biopsy positive	Probable NCC
C34	Epilepsy, fever	Hypodense calcified parieto-occipital brain abscess	Congo Kinshasa	1 serum, 1 brain biopsy	Serum positive/CSF Nd	Serum negative/CSF Nd	Serum Nd/CSF Nd/brain biopsy positive	Probable NCC
C35	Epilepsy, balance disorder, intracranial hypertension	Ventricular dilatation	Madagascar	1 serum, 1 CSF	Serum positive/CSF positive	Serum positive/CSF Nd	Serum Nd/CSF positive	Probable NCC
C36 *	Headaches, epilepsy	1 temporal cystic lesion	No travel, consumption of raw sausage	1 serum	Serum negative/CSF Nd	Serum negative/CSF Nd	Nd	Definitive NCC
C37	Psychiatric and memory disorders, spatial disorientation aphasia	3 IP lesions	Africa	1 serum, 1 CSF	Nd	Serum positive/CSF positive	Serum negative/CSF positive	Definitive NCC
C38	Headaches, neck ache, nausea, vomiting	Multiple intraventricular lesions, ventricular dilatation, foramen of Monro obstruction	Central Africa	1 serum, 1 CSF	Nd	Serum positive/CSF positive	Serum negative/CSF positive	Definitive NCC
C39	Headaches, nausea, vomiting	IP calcifications, hydrocephalus, arachnoiditis	Haiti	1 serum, 2 CSF	Nd	Serum positive /CSF positive	Serum negative/CSF negative then positive	Definitive NCC
C40	Epilepsy, neuralgia, paresthesia	IP calcifications, hydrocephalus, arachnoiditis	Haiti	1 serum, 1 CSF	Nd	Serum positive/CSF positive	Serum negative/CSF positive	Definitive NCC
C41	Epilepsy, headaches, vomiting	2 IP lesions, chronic meningitis	Cape Verde	1 serum, 3 CSF	Nd	Serum positive/CSF positive	Serum negative/CSF positive X3	Definitive NCC
C42	Epilepsy, confusion	Multiple IP lesions, hydrocephalus, vasculitis	Cape Verde	1 serum, 6 CSF	Nd	Serum positive/CSF positive	Serum negative/CSF positive X6	Definitive NCC
C43	Headaches, nausea, memory disorders, dysphasia, spatial disorientation, muscular cysticercosis	IP calcifications, parasagittal meningioma, then hydrocephalus, meningoencephalitis	Madagascar	1 serum, 2 CSF	Nd	Serum positive/CSF positive	Serum negative/CSF positive X2	Definitive NCC
C44	Headaches, epilepsy	4 IP lesions	Cape Verde	1 serum, 1 CSF	Nd	Serum positive/CSF positive	Serum negative/CSF negative	Definitive NCC
C45	Headaches, epilepsy	1 IP lesion	India	1 serum, 1 CSF	Nd	Serum positive/CSF negative	Serum negative/CSF negative	Definitive NCC

* positive brain histology; CSF: cerebrospinal fluid; DRC: Democratic Republic of Congo; IP: intraparenchymal; Nd: not done; C14, C15 were previously published by Šoba et al. 2014 [17]; C36-C45 were previously published by Yera et al. 2011 [10].

## Appendix B. Collaborators Groups

Bernard Bouteille, Marie-Pierre Brenier-Pinchart, Sophie Brun, Estelle Cateau, Eric Caumes, Naima Dahane, Marie-Laure Dardé, Anne Debourgogne, Damien Dupont, Jean-François Faucher, Pierre Flori, Béatrice Garcin, Gilles Gargala, Julie Guellerin, Sandrine Houze, Antoine Huguenin, Arezki Izri, Annie Lannuzel, Muriel Nicolas, Anthony Marteau, Nathalie Pansu, Yvon Sterkers, Feriel Touafek.
